# A case of severe *Plasmodium ovale* malaria with acute respiratory distress syndrome and splenic infarction in a male traveller presenting in Italy

**DOI:** 10.1186/s12936-024-04911-4

**Published:** 2024-04-04

**Authors:** Maria Virginia Tomassi, Alessandra D’Abramo, Serena Vita, Angela Corpolongo, Antonella Vulcano, Tommaso Ascoli Bartoli, Barbara Bartolini, Francesca Faraglia, Emanuele Nicastri

**Affiliations:** National Institute for Infectious Diseases “Lazzaro Spallanzani” IRCCS, Via Portuense 292, 00149 Rome, Italy

**Keywords:** *Plasmodium ovale*, Malaria, Splenic infarction, Acute respiratory distress syndrome

## Abstract

**Background:**

*Plasmodium ovale* malaria is usually considered a tropical infectious disease associated with low morbidity and mortality. However, severe disease and death have previously been reported.

**Case presentation:**

A case of severe *P. ovale* malaria in a healthy Caucasian man with a triangle splenic infarction and clinical progression towards Acute Respiratory Distress Syndrome was reported despite a rapid response to oral chloroquine treatment with 24-h parasitaemia clearance.

**Conclusion:**

*Plasmodium ovale* malaria is generally considered as a benign disease, with low parasitaemia. However, severe disease and death have occasionally been reported. It is important to be aware that occasionally it can progress to serious illness and death even in immunocompetent individuals.

## Background

*Plasmodium ovale* malaria is usually considered a tropical infectious disease associated with low morbidity and mortality. However, severe disease and death have previously been reported [1]. A case of severe *P. ovale* malaria with a triangle splenic infarction and progression towards acute respiratory distress syndrome (ARDS) was reported in a healthy Caucasian man despite a rapid response to oral chloroquine treatment with 24-h parasitaemia clearance.

## Case presentation

**Fig. 1:**
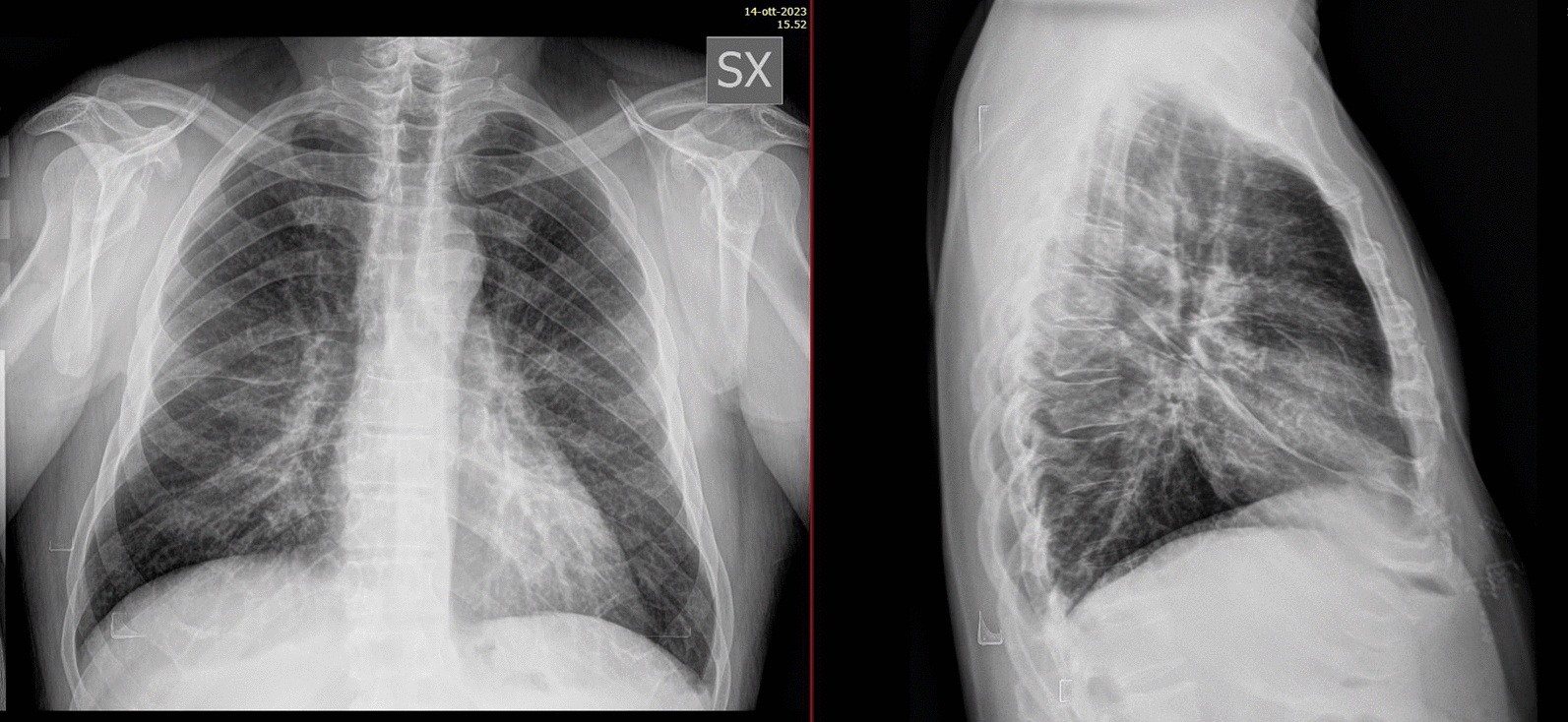
Chest X-ray during at hospital admission

**Fig. 2:**
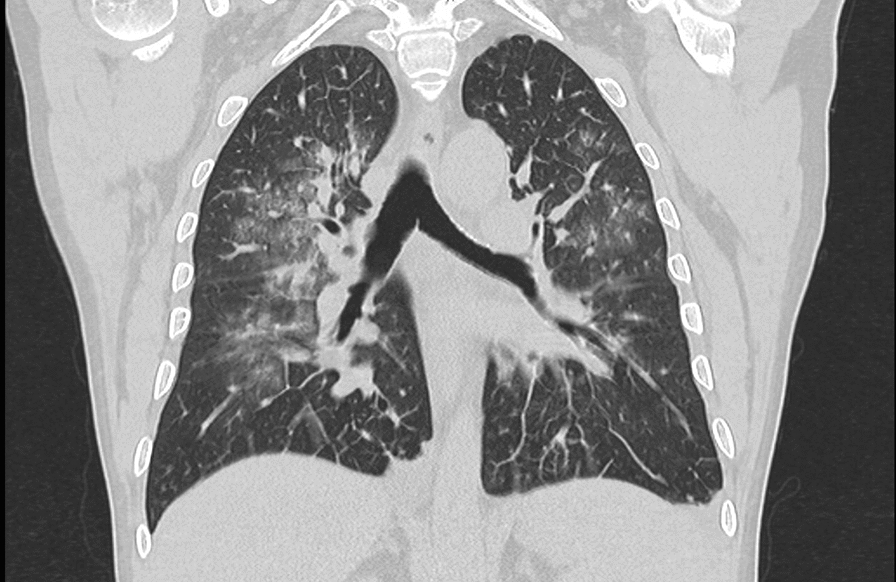
Chest computed tomography during *Plasmodium ovale* malaria showing interstitial bilateral pneumonia with consensual pleural effusion

**Fig. 3:**
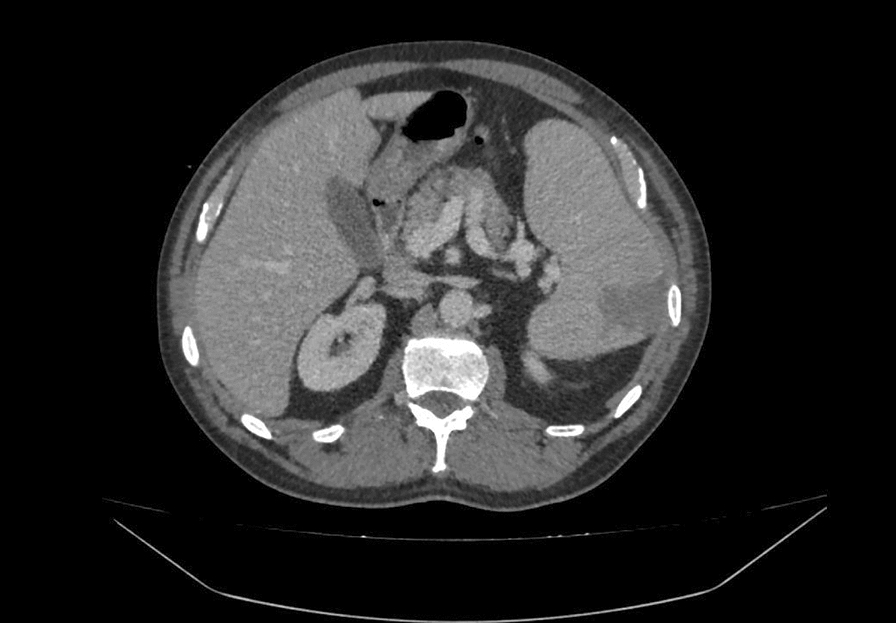
Computed tomography during *Plasmodium ovale* malaria showing splenic infarction

A case of a 4-year-old Caucasian man admitted in the Lazzaro Spallanzani National Institute for Infectious Diseases in Rome, Italy, due to “fever in returning travellers” was reported. At admission, the patient, a healthy man with benign prostatic hyperplasia only, in his medical history, was in good condition. He reported one week of asthenia, headache, night sweats, loss of appetite and, the day before, high fever with syncope. The patient reported travel to South Korea and, two weeks earlier, to Guinea Conakry without taking any anti-malarial chemoprophylaxis. On admission, blood tests showed thrombocytopenia (platelet count 50.000/mmc), C-reactive protein (CRP) 10.6 mg/dl, procalcitonin (PCT) 1.05 ng/ml, aspartate aminotransferase 103 U/L, alanine aminotransferase 67 U/L, gamma-glutamyl transferase 173U/L, with normal renal function and coagulation**.** Pan-malarial rapid test was positive for *non-falciparum* malaria and *Plasmodium vivax* rapid test was negative, thick and thin blood smear were positive and showed the presence of *P. ovale* trophozoites, with a 0.001% parasitaemia (40 parasites/µl). Oral chloroquine, 10 mg/kg as initial dose followed by 10 mg/kg on the second day and 5 mg/kg on the third day, was prescribed. In-house nested-polymerase chain reaction (PCR) confirmed the diagnosis of *P. ovale* excluding mixed infections [2]. *Plasmodium ovale wallikeri* was identified by using a nested PCR followed by 2% agarose gel electrophoresis (a 245 bp band confirmed *P. o. wallikeri*) and verified with amplicon sequencing [3]. Blood PCR for *Leishmania* and Dengue were negative; serology for *Leishmania*, *Schistosoma*, Strongyloides, Dengue, Chikungunya, HCV, HBV, HIV, HEV, Widal-Wright reaction for *Salmonella* and blood cultures were negative. Multiplex Real-Time PCR (Norovirus GII, Rotavirus A, Astrovirus, Norovirus G I, *Cryptosporidium* spp, *Entamoeba histolytica*, *Giardia lamblia*, *Cyclospora cayetanensis*, *Vibrio parahaemolyticus*, *Clostridium difficile* toxin A/B, *Vibrio vulnificus*, Enteropathogenic *Escherichia coli*, *Campylobacter* spp., *Yersinia enterocolitica*, Enterotoxigenic *E. coli* (It/st), Enteroinvasive *E. coli*/*Shigella*. STEC (stx1/stx2), Enteroaggregative *E. coli*, *Salmonella* spp., *Plesiomonas shigelloides*, *Sapovirus*. *Vibrio cholerae* STEC-0157:H7, Adenovirus F40/F41) on stool was negative. Posterior-anterior chest X-ray showed bilateral and diffused peribronchovascular thickening (Fig. 1) and abdominal ultrasound confirmed mild splenomegaly (bipolar diameter 15.5 cm) with homogeneous structure. The day after admission, despite a negative thick smear, the patient’s condition suddenly worsened: he developed dyspnea with a increased respiratory rate of 40 breaths per minute, low peripheral oxygen saturation less than 90%, and acute hypoxaemia confirmed by blood gas analysis. Chest auscultation revealed bilateral crackles in both respiratory phases. The patient started oxygen supplementation with 40% fractional inspired oxygen (FiO2) Venturi mask with improvement of blood gas analysis: PH: 7.47, pCO2: 31, PO2: 104 mmHg with an arterial oxygen tension (PaO2/FiO2 ratio) of 260. Therefore, antibacterial therapy with intravenous ceftriaxone 2 g daily was started. A multiplex Real-Time RT-PCR on sputum was positive for *Staphylococcus aureus*; sputum cultures, quantiferon TB Gold, nasopharynx swab for SARS-CoV-2 and pneumococcal/legionella urine antigen test were negative. After 24 h arterial blood gas analysis showed a reduced oxygen tension (pO2 72, pCo2 29, PaO2/FiO2 ratio of 180), Continuous positive airway pressure (CPAP) by using 40%FiO2 Boussignac mask and 5 mmhg positive end-expiratory pressure (PEEP) was started. A chest and abdomen computed tomography scan with intravenous contrast showed interstitial ground glass opacities involving all right and left lobes with early parenchymal lung consolidation, bilateral pleural and pericardium effusion (Fig. 2) and in the abdomen there was hypodense area with triangular morphology at splenic site (Fig. 3). The findings of the CT scan lay for cardiogenic oedema and triangle splenic infarction. The patient had no abdominal pain. Patient fluid balance was initially positive. Intravenous furosemide (20 mg every 12 h) and piperacillin/tazobactam (4.5 g every 8 h), oral doxycycline (100 mg every 12 h), enoxaparin 40 mg subcutaneous daily were started**.** In the following days, the patient gradually improved with decreasing the needs of oxygen until the definitive ending on day 15 since admission. Oral primaquine (30 mg daily for 14 days) was given after confirming a normal level of glucose-6-phosphate dehydrogenase activity in erythrocytes. The patient was discharged in good clinical condition after 18 days of hospitalization, with normal blood tests. Two weeks later at follow-up visit, blood tests were repeated with unremarkable results and no evidences of sequelae or relapse were reported.

## Discussion and conclusion

A severe *P. ovale* malaria with a triangle splenic infarction and clinical progression towards ARDS was described despite a rapid response to chloroquine with fast parasitaemia clearance. Suddenly, with no underlying risk factors for severe disease, patient’s condition worsened with acute respiratory failure with CPAP-oxygen supplementation. Severe malaria is usually associated with *Plasmodium falciparum* infection. *Plasmodium vivax* and *Plasmodium knowlesi* are recognized as possible causes of severe disease. In contrast, *P. ovale* malaria is generally considered a benign disease, with low parasitaemia [4]. Two sympatric species of *P. ovale* occur globally: *P. ovale curtisi* (classic type) and *P. ovale wallikeri* (variant type) [5]. Rare case reports of complicated *P. ovale* malaria are reported and only in few of them *P. ovale* sub-species have been identified. ARDS is the prevalent severe condition described [6, 7]; anecdotal cases of splenic rupture [8] and a single case of splenic infarction are described previously [9]. A recent case report describes a haemophagocytic lymphohistiocytosis secondary to *P. ovale wallikeri* infection [10]. The 2023 WHO Guidelines for uncomplicated *P. ovale* malaria recommend either an artemisinin-based combination therapy (ACT) or chloroquine, in areas with chloroquine-susceptible infections. In areas with chloroquine-resistant infections, adults and children with uncomplicated *P. ovale* malaria should be treated with ACT [11]. Chloroquine is the only option recommended by CDC [12]. The pathogenesis of *P. ovale* severe infection has not been well established. It is well known that ARDS in *P. falciparum* malaria is caused by increased pro-inflammatory process with unbalanced endothelial permeability; intravenous over hydration, sequestration of red cells, and disseminated intravascular coagulation are all other likely determinants of severe ARDS [13]. In this case, the patient presented a positive fluid balance, thrombocytopenia and both increased CRP and PCT [14]. Chloroquine-resistance was excluded because of the early negative thick smear. Although chloroquine is known to have an anti-inflammatory modulation, inhibiting the production of cytokines such as TNF, IL-1β and IL-6 [15], in this case it did not seem to prevent the cascade of the inflammatory phenomenon leading to respiratory distress. Regarding splenic infarction, this is the second case described in the literature due to *P. ovale* malaria [16], likely to be part of a *Plasmodium*-driven coagulation and proinflammatory disbalance leading to an increased adhesion of malaria-infected red blood cells (iRBCs) to endothelial cells, with rosetting of iRBCs and non-iRBCs, and to splenic cell hyperplasia [9, 17]. Splenic infarction has rarely been reported as a complication of *P. falciparum* or *P. vivax* malaria. This event is likely to be underdiagnosed and underreported. Indeed, patients with splenic infarction can be asymptomatic or present only mild symptoms and, in resource-limited settings, abdomen ultrasound or computer tomography imaging could be not available [9].

In conclusion, more studies are needed to better define the pathogenesis of severe malaria due to *P. ovale* and the clinical implication with its *wallikeri or curtisi* sub-species. *Plasmodium o. wallikeri* may be more frequent in males and Caucasians. Moreover, *P. o. wallikeri* could have greater pathogenicity with a deeper thrombocytopenia, a higher INR and shorter latency than *P. o. curtisi* [18]. Severe cases can be seen in both species but in a French cohort, although the difference was not statistically significant, *P. o. wallikeri*–infected patients were 5 times more frequently hospitalized in intensive care or intermediate care than *P. o. curtisi*–infected patients [19]. Furthermore, severe cases of *P. ovale* malaria could be under-reported due to misclassification by rapid diagnostic tests poor sensitivity for *P. ovale* species [20], to diagnostic difficulties in the detection of a low parasitaemia and in the differential species detection between *P. ovale* and *P. vivax*, when PCR is not available. Finally, host–pathogen risk factor, genetic determinants, comorbidities and predictive markers of clinical progression to severe malaria and acute respiratory distress in *P. ovale* are unclear.

## Data Availability

Not applicable.

## Abbreviations

CRP: C-reactive protein
PCT: Procalcitonin
PCR: Polymerase chain reaction
FiO2: Fractional inspired oxygen
PaO2: Arterial oxygen tension
CPAP: Continuous positive airway pressure
PEEP: Positive end-expiratory pressure
ARDS: Acute respiratory distress syndrome
WHO: World Health Organization
ACT: Artemisinin-based combination therapy
CDC: Centers for disease control and prevention
iRBCs: Malaria-infected red blood cells
